# Documentation of Advance Care Planning in Early Phase Cancer Clinical Trials: An Australian Single-Centre Experience

**DOI:** 10.3390/cancers17223655

**Published:** 2025-11-14

**Authors:** Nancy Huang, Joseph Descallar, Samuel Vo, Su Saint Lee, Kate Wilkinson, Aflah Roohullah, Adam Cooper, Victoria Bray, Wei Chua, Danielle Ní Chróinín, Abhijit Pal

**Affiliations:** 1 Liverpool Cancer Therapy Centre, Liverpool Hospital, Liverpool, NSW 2170, Australia; 2 Faculty of Medicine, Health and Human Sciences, Macquarie University, Sydney, NSW 2109, Australia; 3 Ingham Institute for Applied Medical Research, Liverpool, NSW 2170, Australia; 4 School of Clinical Medicine, University of New South Wales Sydney, South West Sydney Clinical Campus, Liverpool, NSW 2170, Australia; 5 Liverpool Clinical School, Western Sydney University, Liverpool, NSW 2170, Australia

**Keywords:** advance care planning, clinical trial, documentation, medical records, neoplasms

## Abstract

Background/Objectives: Patients with incurable cancers enrolled in early phase clinical trials often face uncertainty about prognosis, yet advance care planning (ACP) is frequently delayed. The objective of this study was to assess the documentation of ACP discussions among patients enrolled in early phase oncology trials. Methods: We conducted a retrospective review of electronic medical records for all adults enrolled in early phase clinical trials at a single Australian institution (2012–2021). Data included time from metastatic diagnosis to first ACP discussion, clinical and sociodemographic factors, triggers for discussion, and clinician specialty. Results: Among 170 patients (58% male; median age 65 years), ACP documentation was identified in 109 (64%). ACP was most often initiated within the final year of life (73.8%), with a median interval of 23.5 months from metastatic diagnosis to first documentation. Common triggers were disease progression (39.6%) and hospital admission (37.8%). Discussions were typically led by the treating oncologist or trials specialist (43%) and palliative care physician (37.8%). The most frequently documented topic was the limitations of invasive care such as intubation (60%). Conclusions: ACP documentation was present in two-thirds of patients enrolled in early phase clinical trials, typically late in the disease trajectory. Integrating structured, earlier ACP discussions into oncology pathways would improve alignment of care with patient goals and enhance end-of-life care.

## Simple Summary

Many people with advanced cancer take part in early phase clinical trials to access new treatments. These patients face complex medical decisions, yet conversations about their future care wishes (also called advance care planning) are often delayed. This study reviewed the medical records of cancer patients who participated in an early phase clinical trial to understand when and how these important discussions occurred. We discovered that most conversations about future care happened late, usually in the last year of life, and were often triggered by worsening illness or hospital admission. By identifying how and when these talks take place, this research highlights opportunities to start them earlier, with the goal of ensuring that future medical care is aligned with each patient’s individual values and priorities.

## 1. Introduction

Advance care planning (ACP) is a key component of high-quality end-of-life care for people with advanced cancer. International guidelines advocate for early initiation of ACP [1], which is defined as a process that enables individuals to articulate and document their personal values, goals, and preferences for future medical care [2].

ACP provides meaningful clinical, psychological, and practical benefits for patients and their families. It enhances patients’ understanding of their illness without reducing hope or increasing anxiety, improves psychological well-being, and increases the likelihood of patients dying in their preferred place, which is often home [3,4,5]. For families and carers, ACP reduces stress, anxiety, and depression [6]. It also encourages the completion of advance care directives and legal documents, the appointment of substitute decision-makers [7], and earlier referral to palliative care [8]—factors that have been associated with fewer hospital presentations, admissions, and deaths [9].

Despite the well-recognised advantages, the uptake of ACP in Australia remains low, with limited evidence regarding the optimal timing of ACP in cancer care. A national survey reported that only a small minority of Australians had completed a formal advance care directive [10], and chart audits reveal even lower rates of documentation [11], underscoring the need for accurate and accessible ACP records. Consistent documentation is critical for ensuring continuity of care, supporting shared decision-making, and providing legal clarity when patients lose capacity. In Australia, various states have legislated ACP forms that permits documentation of patient preferences for medical care but there is no unifying legal document. This in combination with the use of different electronic medical record (EMR) systems across different health districts contributes to fragmented documentation and missed opportunities for meaningful discussion.

Cancer patients participating in early phase clinical trials (EPCT) represent a group for whom timely ACP is particularly important. These individuals have stage IV incurable disease and limited life expectancy following exhaustion of standard lines of treatment [12,13,14], and are pursuing experimental therapies that rarely achieve durable responses [15,16]. In addition, therapeutic misconception is widespread amongst the EPCT population [17]; while there have been cases of individual patients deriving a prolonged benefit from EPCT, the vast majority still have a poor prognosis. The primary goals of EPCT are to determine maximal safe dosing and toxicity profiles [18], which may conflict with patient expectations for therapeutic benefit [19]. Yet, discussions about prognosis and alternative pathways such as best supportive care are often absent or incomplete [20]. In one study, 59% of patients enrolled in EPCT expressed a desire to discuss ACP with their physicians [21]. While this figure may seem encouraging, over half of the eligible patients declined to complete the survey [21], reflecting an underlying resistance to ACP that merits further exploration. Collectively, these findings underscore the multifaceted barriers that hinder ACP conversations in clinical trial settings.

Given these complexities, the optimal timing of ACP initiation in patients with advanced cancer remains uncertain. Initiating ACP early may result in outdated preferences, whereas late discussions risk rushed or reactive decision-making [22]. Even among oncologists, there is limited consensus on what constitutes “early” ACP [23]. Understanding current practice patterns and identifying opportunities to promote earlier, well-documented discussions are therefore essential to improving patient-centred care at the end of life.

The primary objective of this study was to examine the documentation of ACP discussions among patients with metastatic solid organ malignancies enrolled in EPCT at Liverpool Hospital.

## 2. Materials and Methods

### 2.1. Study Design and Objectives

Retrospective medical record review is a well-established method for assessing the prevalence of end-of-life documentation [24]. We conducted a retrospective review of electronic medical records (EMR) for patients treated in the Liverpool Hospital Phase I Clinical Trials Unit between 2012 and 2021. Specific objectives were to describe patient clinicopathological and clinician characteristics and discussion settings. Overall, this study sought to explore how health professionals within the district approach ACP in EPCT patients.

The study was undertaken within the South Western Sydney Local Health District, which includes Liverpool, Bankstown-Lidcombe, Campbelltown, and Fairfield hospitals. Liverpool Hospital hosts a major tertiary oncology centre with an established EPCT unit that receives both internal and external referrals. Eligible participants were adults with metastatic solid organ malignancy who had been enrolled in an EPCT at Liverpool Hospital between 2012 and 2021.

### 2.2. Data Collection

Data were obtained from two primary sources: the EMR system and the district cancer service oncology information system MOSAIQ (Elekta, version 2.63). Clinical documentation from physicians, nursing, and allied health professionals was reviewed by one investigator, with every 20th record cross-checked by a second investigator for consistency and accuracy. Data cut-off was defined as the late date of follow up for which all enrolled participants had at least 6 months of follow up.

Baseline patient characteristics collected included age, sex, country of birth, language preference and Eastern Cooperative Oncology Group (ECOG) performance status [25]. Cancer-related variables included tumour type, date of metastatic diagnosis, prior lines of systemic therapy, and survival outcomes. For patients with documented ACP discussions, data were collected on the date and context of the first discussion, location, precipitating events (e.g., disease progression, hospital admission), clinician specialty, and ECOG status at the time of discussion.

ACP was defined as any documented process that reflected a patient’s values, goals, or preferences regarding future medical care. The specific components of ACP (e.g., limitations of care, such as resuscitation or intubation preferences) were also recorded.

### 2.3. Statistical Analysis

We employed simple descriptive analyses with frequencies and proportions. Logistic regression was used to analyze predictor variables with having an ACP. Patients were followed from the date of clinical trial enrolment to their final study date or their death where applicable. *p*-value < 0.05 was considered statistically significant. Statistical analysis was performed in SAS enterprise guide version 8.5.

## 3. Results

Between 2012 and 2021, 170 patients with metastatic disease were enrolled in EPCT and eligible for inclusion.

### 3.1. Patient Characteristics

A total of 170 patients were included in the analysis, of whom 99 (58%) were male (Table 1). The median [21] age at trial enrolment was 65 years (range 31–88 years) and 43.5% of patients were born outside of Australia. At enrolment, all patients had an Eastern ECOG performance status of 0–1. The majority (83%) had received two or more prior lines of systemic therapy. The most common tumour types were lower gastrointestinal malignancies (26.5%), followed by upper gastro-intestinal cancers (21.2%). At the time of data cut-off, 157 patients (91%) were deceased.

### 3.2. Patient Characteristics Between Those with and Without ACP

In the study population, 109 patients (64%) had documentation of ACP (Table 2). Patients with an ECOG of 1 had a higher likelihood of having documentation of ACP (odds ratio 2.03, 95% CI 1.03–4.30, *p*-value 0.04). Patients with ≥3 lines of treatment also had a higher likelihood of having documentation of ACP (odds ratio 2.32, 95% 1.2–4.49, *p*-value 0.01).

ACP documentation did not differ by gender, age (≥65 versus <65 years of age), country of birth, language preference or co-morbidities (Table 2).

### 3.3. Timing and Details of ACP Discussions

ACP discussions were documented for 109 patients (64%) and were most often initiated during the patient’s final year of life (73.4%, n = 80). The median interval from diagnosis of metastatic disease to first ACP documentation was 23.5 months.

The most common triggers for initiating ACP discussions were disease progression (40.5%, n = 45) and hospital admission (37.8%, n = 42) (Table 3). Discussions were most frequently led by the treating medical oncologist, including the primary or EPCT specialist (43%, n = 48), or a palliative care physician (37.8%, n = 42).

Communication of ACP outcomes to the patient’s nominated general practitioner was inconsistently documented, with evidence of a discharge or clinic letter describing the discussion in only 34.2% (n = 38) of cases.

There was considerable variation in the content of ACP discussions. The most frequently addressed element was the limitation of invasive interventions, such as intubation or resuscitation (60%, n = 67).

## 4. Discussion

In this retrospective study of patients with metastatic cancer referred for EPCT at a large Australian institution, one in three patients had no electronic documentation suggesting an ACP discussion had occurred. Despite the poor prognosis associated with this cohort, ACP discussions typically occurred late in the disease trajectory, if at all.

The median interval from diagnosis of metastatic disease to the first documented ACP discussion was 24 months, highlighting the ongoing challenge of initiating ACP early in the patient journey. The optimal timing for ACP is complex and often influenced by patient readiness, family dynamics, health literacy, and the unpredictable course of illness. Although ACP has traditionally been initiated when curative options are exhausted [26] or when the malignancy becomes incurable [27], there is increasing recognition that early and iterative discussions during routine oncology care enhance patient-centred decision-making [28]. A 2020 study reported that almost half of patients would prefer to begin ACP discussions before their cancer diagnosis [28], underscoring the importance of proactive engagement.

Our study demonstrated that patients with an ECOG performance status of 1, or those who had received three or more lines of systemic therapy, were more likely to have documentation of ACP compared with patients with an ECOG of 0 or fewer than three treatment lines. This finding is consistent with expectations, as poorer performance status and greater treatment exposure typically indicate higher tumour burden or more treatment-resistant disease, circumstances in which ACP discussions are more likely to occur. The absence of a statistically significant difference in ACP documentation between English and non-English speaking groups was unexpected. However, this may reflect the characteristics of a selective trial population, in which participants are more engaged in communication and decision-making processes than the average patient population.

In our cohort, ACP was most commonly triggered by acute clinical deterioration, such as disease progression or hospital admission. This suggests that clinicians may rely on external events to initiate these conversations rather than embedding ACP as a routine element of care. Existing research indicates that patients prefer ACP to be initiated by their doctors, likely reflecting underlying power dynamics within the clinician–patient relationship [29]. Training programs have been shown to enhance physicians’ understanding of ACP’s relevance, as well as their confidence and willingness to initiate discussions [30]. Taken together with our findings, this suggests that structured education and training may help overcome key physician-related barriers to ACP.

Encouragingly, the majority of ACP discussions in our study involved the patient directly (86.5%), and a family member was present in more than half of cases. Family involvement in end-of-life discussions is consistently valued by people with cancer [27]. Patients also tend to prefer ACP discussions with clinicians who know them well and possess the communication skills required to manage such nuanced conversations [29]. One study found that patients favoured their primary care provider over their oncologist or surgeon, attributing this preference to greater trust, familiarity, and stronger interpersonal connections with their general practitioner [28]. However, communication with primary care providers was often lacking; only one-third of ACP discussions were documented in correspondence to general practitioners. This gap risks fragmented continuity of care, particularly in emergencies. Strengthening the integration between oncology and primary care would help ensure that patient wishes are accessible across different care settings.

Both medical oncologists (43%) and palliative care physicians (38%) were equally involved in ACP initiation. Palliative care input for EPCT participants is essential for managing symptoms and treatment toxicities and has been shown to improve both patient quality of life and trial retention [31]. Our findings reinforce that palliative care involvement and clinical trial participation should not be viewed as mutually exclusive, but rather as complementary aspects of holistic cancer care.

The most frequent ACP topic documented was limitation of invasive care (e.g., resuscitation or intubation preferences). This narrow focus contrasts with literature showing that patients also value discussions around decision-making authority, symptom management, and maintaining consciousness [27]. The discrepancy likely reflects the limitations of retrospective data, where documentation may not capture the full scope or quality of patient-centred communication.

Documentation practices in our cohort were inconsistent. Although most ACP discussions were recorded in electronic medical records (59.5%), less than half were reflected in the oncology database or clinic correspondence. This inconsistency may reflect workflow habits, where free-text notes are entered into MOSAIQ but not consistently transferred to formal letters. Failure to communicate ACP outcomes beyond the treating institution could lead to misaligned care in community or emergency settings, particularly if treating clinicians are unaware of prior decisions. Our study highlights the real-world challenges of communication and documentation of patients’ wishes in complex healthcare systems.

Our study’s strengths include the relatively large cohort, inclusion of a culturally diverse population that is typically under-represented in cancer care research [32], and focus on a niche group—patients enrolled in EPCT—where ACP is often overlooked. In terms of exploring the impact of cultural diversity on documentation of ACP, we focused on country of birth and language preference, acknowledging that there is enormous heterogeneity in how a culturally and linguistically diverse population is defined in epidemiological research [33]. By combining country of birth and language preference, our study captures the ACP experiences of different generations of immigrants to Australia who have participated in EPCT. Given the cultural and ethnic diversity of the South Western Sydney region, future studies should explore more nuanced indicators of cultural background and health literacy to understand barriers to ACP participation.

We acknowledge several limitations. As a retrospective review, our study relied on the accuracy and completeness of existing documentation; paper records were not included, and resuscitation plans may have been omitted if not digitised. Data from other hospitals or out-of-district presentations were unavailable, potentially underestimating ACP activity. Moreover, documentation may not fully reflect the occurrence or quality of ACP discussions, as there are individual variations in documentation. Finally, the examination of only a single trial centre and focus on clinical trial participants will limit the application of results to broader oncology populations.

### Implications for Clinical Practice

Our findings highlight a clear opportunity for earlier and more consistent integration of ACP in the care of patients with advanced cancer, particularly those enrolled in early-phase clinical trials. The increasing complexity of investigational therapies, including immunotherapies and targeted agents, demands nuanced conversations about prognosis, goals of care, and evolving treatment expectations.

Recent improvements in EPCT outcomes—including higher response rates [34], reduced treatment-related mortality [18], and greater use of non-cytotoxic agents [35]—are redefining the traditional view of EPCT as purely “last-line” options. Accordingly, ACP should not be deferred until all treatment options are exhausted, but integrated as a continuous, evolving process throughout the patient’s cancer journey.

The introduction of voluntary assisted dying (VAD) legislation across several Australian states adds a further dimension to ACP. Clinicians must distinguish ACP—which guides future care when capacity is lost—from VAD, which only applies to individuals with decision-making capacity [36]. As VAD becomes part of the Australian end-of-life landscape, healthcare systems must ensure clinicians are equipped with clear guidance, training, and support to navigate these discussions ethically and sensitively within ACP frameworks.

## 5. Conclusions

In this study of patients with metastatic cancer enrolled in EPCT, one in three had no documented advance care plan, and when present, discussions often occurred late in the disease course. ACP was usually prompted by clinical deterioration rather than integrated into routine care. These findings highlight barriers at clinician and institutional levels, emphasising the need for greater awareness and timely documentation. Embedding ACP within oncology and clinical trial workflows, supported by education and improved communication systems, could facilitate earlier, patient-centred discussions and ensure that future care aligns with individual values and preferences.

## Figures and Tables

**Table 1:** Patient demographics.

	n	Percent
All patients	170	100
Sex, male	99	58
Age	Median age 65 (range 31–88)	
Country of birth		
Australia	96	56.5
Asia	30	17.6
Europe	31	18.2
Other	13	7.6
Language preference		
English	138	81.2
Other	32	18.8
ECOG PS at time of trial enrolment		
0	53	31.2
1	117	68.8
Co-morbidities by Charles Co-morbidity Index		
0	21	12.4
1	42	24.7
2	34	20
>3	73	42.9
Previous lines of treatment		
0	6	3.5
1	23	13.5
2	58	34.1
3	44	25.9
≥4	39	22.9
Tumour type		
Genito-urinary	33	19.4
Upper gastro-intestinal	36	21.2
Lower gastro-intestinal	45	26.5
Lung	6	3.5
Gynaecological	18	10.6
Head and neck	8	4.7
Unknown primary	1	1
Breast	8	4.7
Other	15	8.8

**Table 2:** Comparison of patient characteristics between those with and without ACP.

	ACP		No ACP			Multivariable Model			
	n	%	n	%	*p*-Value	Odds Ratio	Lower 95% CI	Upper 95% CI	*p*-Value
Sex									
Male	64	64.7	35	35.4	0.8727				
Female	45	63.4	26	36.6					
Age									
≥65	60	65.9	31	34.1	0.6328				
<65	49	62	30	38					
Country of birth									
Australia	56	58.3	40	41.7	0.0787				
Other	53	71.6	21	28.4					
Preferred language									
English	86	62.3	52	37.7	0.4137				
Other	23	71.9	9	28.1					
ECOG									
1	82	70.1	35	29.9	0.0243	2.03	1.03	4.03	0.0424
0	27	50.9	26	49.1					
Lines of treatment									
≥3	62	74.7	21	25.3	0.0064	2.32	1.2	4.49	0.0125
0–2	47	54	40	46					
Co-morbidities									
≥3	46	63.9	26	36.1	0.1286				
0–2	62	63.9	35	36.1					

**Table 3:** Details of ACP.

ACP Details	n	Percent
Preceding event prior to ACP		
Disease progression	45	40.5
Hospital admission	42	37.8
Physician interaction	24	21.6
Patient/relative interaction	11	9.9
Unclear	11	9.9
Patient/carer involvement		
Patient	96	86.5
Relative	58	52.3
Other	2	1.8
Location of discussion		
Clinic	45	40.5
Ward	29	26.1
Emergency department	17	15.3
Other	20	18
Documentation		
Clinic letter	24	21.6
Electronic medical record	66	59.5
MOSAIQ (Cancer Database)	46	41.4
Details of ACP		
Identification of formal Enduring Guardian	4	3.6
Personal beliefs	11	9.9
Preference for end-of-life care	27	24.3
Directions about medical care	67	60.4
Other	34	30.6
Sharing of Decision with General Practitioner *		
Yes	38	34.2
No	73	65.8
Clinicians		
Female	72	62.2
Male	39	35.1
Clinicians’ speciality		
Medical oncology	48	43.2
Palliative care	42	37.8
Emergency Department	10	9
Other	11	9.9

* Through documentation such as that of a discharge summary or clinical letter.

## Data Availability

The original contributions presented in this study are included in the article. Further inquiries can be directed to the corresponding author(s).

## Abbreviations

The following abbreviations are used in this manuscript:

ACP	Advance care planning
ECOG	Eastern Cooperative Oncology Group
EPCT	Early phase clinical trials
VAD	Voluntary assisted dying
